# Adequacy of an Altitude Fitness Program (Living and Training) plus Intermittent Exposure to Hypoxia for Improving Hematological Biomarkers and Sports Performance of Elite Athletes: A Single-Blind Randomized Clinical Trial

**DOI:** 10.3390/ijerph19159095

**Published:** 2022-07-26

**Authors:** Diego Fernández-Lázaro, Juan Mielgo-Ayuso, Gema Santamaría, Eduardo Gutiérrez-Abejón, Carlos Domínguez-Ortega, Sandra María García-Lázaro, Jesús Seco-Calvo

**Affiliations:** 1 Department of Cellular Biology, Genetics, Histology and Pharmacology, Faculty of Health Sciences, Campus of Soria, University of Valladolid, 42003 Soria, Spain; gemasantgo@gmail.com (G.S.); cdominguezo@saludcastillayleon.es (C.D.-O.); 2 Neurobiology Research Group, Faculty of Medicine, University of Valladolid, 47005 Valladolid, Spain; 3 Department of Health Sciences, Faculty of Health Sciences, University of Burgos, 09001 Burgos, Spain; 4 Pharmacological Big Data Laboratory, Faculty of Medicine, University of Valladolid, 47005 Valladolid, Spain; egutierreza@saludcastillayleon.es; 5 Pharmacy Directorate, Castilla y León Health Council, 47007 Valladolid, Spain; 6 Centro de Investigación Biomédica en Red de Enfermedades Infecciosas (Group CB21/13/00051), Carlos III Institute of Health, 28029 Madrid, Spain; 7 Hematology Service of Santa Bárbara Hospital, Castile and Leon Health Network (SACyL), 42003 Soria, Spain; 8 Department of Surgery, Ophthalmology, Otorhinolaryngology, and Physiotherapy, Faculty of Health Sciences, Campus of Soria, University of Valladolid, 42003 Soria, Spain; sandramaria.garcia@uva.es; 9 Physiotherapy Department, Institute of Biomedicine (IBIOMED), Campus of Vegazana, University of Leon, 24071 Leon, Spain; dr.seco.jesus@gmail.com; 10 Psychology Department, Faculty of Medicine, Basque Country University, 48900 Leioa, Spain

**Keywords:** hypoxia, athletes, blood biomarkers, sports performance, safety profile, altitude training

## Abstract

Athletes incorporate altitude training programs into their conventional training to improve their performance. The purpose of this study was to determine the effects of an 8-week altitude training program that was supplemented with intermittent hypoxic training (IHE) on the blood biomarkers, sports performance, and safety profiles of elite athletes. In a single-blind randomized clinical trial that followed the CONSORT recommendations, 24 male athletes were randomized to an IHE group (HA, *n* = 12) or an intermittent normoxia group (NA, *n* = 12). The IHE consisted of 5-min cycles of hypoxia–normoxia with an FIO_2_ of between 10–13% for 90 min every day for 8 weeks. Hematological (red blood cells, hemoglobin, hematocrit, hematocrit, reticulated hemoglobin, reticulocytes, and erythropoietin), immunological (leukocytes, monocytes, and lymphocytes), and renal (urea, creatinine, glomerular filtrate, and total protein) biomarkers were assessed at the baseline (T1), day 28 (T2), and day 56 (T3). Sports performance was evaluated at T1 and T3 by measuring quadriceps strength and using three-time trials over the distances of 60, 400, and 1000 m on an athletics track. Statistically significant increases (*p* < 0.05) in erythropoietin, reticulocytes, hemoglobin, and reticulocyte hemoglobin were observed in the HA group at T3 with respect to T1 and the NA group. In addition, statistically significant improvements (*p* < 0.05) were achieved in all performance tests. No variations were observed in the immunological or renal biomarkers. The athletes who were living and training at 1065 m and were supplemented with IHE produced significant improvements in their hematological behavior and sports performance with optimal safety profiles.

## 1. Introduction

Recently, the sports performance levels in elite competitions have increased considerably and the differences between the results among the top positions have become smaller and smaller. Therefore, coaches and elite athletes of different specialties often combine additional preparation strategies (biological, pharmacological, mechanical, etc.) with their usual training programs in the hope of improving their physiological responses (muscular, blood, cardiovascular, respiratory, endocrine–metabolic, etc.), which could induce improvements in their sports performance, especially for endurance sports, such as athletics, cycling, and triathlon [1]. One strategy that is employed is altitude training (in any of its forms), which has been endorsed by the world’s top athletes. Continuous exposure to hypoxia, which is typical of altitude training, triggers a series of physiological responses and adaptations that are beneficial to sports performance when the arterial saturation of oxygen (SaO_2_) is lower than 90% (Table 1) [2].

To increase the likelihood of achieving beneficial physiological adaptations, especially for hematological biomarkers, it has been reported that it is necessary for athletes to be exposed to an actual altitude of at least 2000–2500 m for at least 90% of daytime hours for at least 4 weeks [3]. However, these extended stays at high altitudes could have negative impacts on the intensity of training and affect sports performance [4]. To counteract these disadvantages of living and training at altitude, new instruments have been proposed that can simulate the effects of altitude training [5]. The simulated altitude training strategies that are most commonly used among elite athletes are intermittent hypoxia exposure (IHE) and intermittent hypoxic training (IHT). Both conditions are applied using reduced oxygen (O_2_) breathing instruments (i.e., mixed gas masks, chambers, tents or rooms, and reduced O_2_ breathing devices) [6]. IHE alternates phases of hypoxia and normoxia. IHT consists of continuous or intervallic training under hypoxic conditions (normobaric or hypobaric) [2]. IHT programs appear to be much more useful than IHE programs in terms of achieving physiological adaptations (especially hematological adjustments) and sports performance [7]. However, IHT can cause an increase in wear and tear, which induces notable muscular catabolism, greater fatigue, and alterations in immunological biomarkers that trigger immunosuppression compared to training under normoxic conditions [8,9,10,11]; therefore, the recovery time between workouts has to be longer, which can alter classic training systems [12].

Although combining the use of IHE with physical training at altitude is not common [13], it could potentially be a suitable alternative for achieving the benefits and minimizing the risks of IHT. To our knowledge, only one study has evaluated living and training at low altitude (825 m) with supplementary IHT sessions [14] and no studies have investigated the effects of living and training at medium altitude (1065 m) with supplementary IHE sessions. In view of this situation, we decided to evaluate the differences between the hematological biomarkers and sports performance of professional athletes who were living and training continuously at altitude (1065 m) and completed an 8-week program of normobaric IHE and a group of professional athletes who completed the same training and were living at altitude but were not exposed to normobaric IHE. In addition, the safety profiles of the normobaric IHE were evaluated using immunological and renal biomarkers. We hypothesized that IHE would substantially improve performance because of the potential benefits for hematological biomarkers that could be induced without causing harmful effects on the athletes.

## 2. Material and Methods

### 2.1. Design and Participants

Twenty-four (*n* = 24) professional male athletes (middle- and long-distance) participated in a blinded randomized clinical trial to evaluate the effects of 8 weeks of IHE on different hematological parameters (white blood cells (WBC), monocytes (MON), lymphocytes (LYN), red blood cells (RBC), hemoglobin (Hb), hematocrit (Hct), reticulocyte hemoglobin (RET-Hb), reticulocytes (RET), and erythropoietin (EPO)) and biomarkers for renal performance (urea, creatinine (Cr), glomerular filtration rate CDK-EPI (GFR CDK-EPI) and total protein (TP)) and sports performance (strength, speed, aerobic power, and anaerobic power).

During the study, the recommendations of the Consolidated Standards of Reporting Trials (CONSORT) group for the conduct of randomized parallel group trials were followed (Appendix A) [15]. The sample size estimation was performed using the G* Power 3.1.97 statistical power analysis program (University of Dusseldorf, Dusseldorf, Germany; available at https://es.freedownloadmanager.org/Windows-PC/Gpower-GRATIS.html) (accessed on 1 June 2022) [16]. To estimate the number of subjects that was needed to evaluate the differences between the independent groups, we followed the approach that was proposed by Calvo-Lobo et al. [17] for studies with small sample sizes. Thus, we considered the differences between the values that were found in the pilot study, which was conducted (*n* = 10) using two groups of ten patients with an α error of 0.05 and a β error of 0.20. This calculation indicated that at least 12 subjects were needed in each group (*n* = 24), considering a possible dropout rate of 20%.

The participants underwent cardiopulmonary and electrocardiographic examinations and were asked to complete a medical questionnaire before entering the study. The exclusion criteria included any pre-existing physical health problems, alcohol consumption or the use of illegal drugs or substances (stimulants, blood derivatives, anabolic agents, etc.) that could alter hematological or renal responses or sports performance. There were no reported injuries before or during the study as injured participants were ruled out by their history and clinical examinations. A dietitian–nutritionist developed an individual diet for each participant that was based on the pre-established nutritional, energy, and macronutrient guidelines for adequate sports performance and the participant’s training volume and training load [18]. All athletes performed the same training sessions during the precompetitive period (Table 2). The training program was supervised by a coach from the Royal Spanish Athletics Federation, who had more than 30 years of experience with athletes who achieved international success in long-distance and middle-distance events.

### 2.2. Experimental Protocol

The athletes in the IHE intervention (HA) group completed a 90-min session of IHE every day for 8 weeks, which took place 1 h after morning training and during which they were seated at rest. The IHE was administered at a ratio of 5 min under hypoxic conditions followed by 5 min of normoxic conditions. Normobaric hypoxic gas was administered using a GO_2_ altitude hypoxia device (Biomedtech, Victoria, Australia). According to the manufacturer’s instructions, the O_2_ concentration was progressively reduced to allow for sufficient adaptation time (Table 3).

The athletes in the control (NA) group also completed a 90-min session of simulated therapy every day for 8 weeks, which took place 1 h after morning training. To perform the simulated therapy, the GO_2_ altitude hypoxia device was used (as for the HA group) but performed 5-min cycles of normobaric normoxic air and normoxic room air.

Both groups had their SaO_2_ levels constantly measured, either automatically using the GO_2_ altitude hypoxia device or manually using a finger pulse oximeter (INVIPOX LTD800, Dimer, Vizcaya, Spain). None of the participants were acclimatized or previously exposed to hypoxia, except for living in Soria (1065 m). The participants were from provinces in Spain that are at lower altitudes than Soria and they had not completed any training camps at altitude in the previous 6 months.

### 2.3. Anthropometry

Skinfolds (mm) (tricipital, bicipital, abdominal, suprailiac, subscapular, iliac crest, front thigh, and calf) were analyzed using a Holtain^®^ skinfold caliper (Crosswell, Crymych, Pembs., SA41 3UF, UK) with a precision of within 0.5 mm. In order to obtain more information about body fat, the sums of six skinfolds (mm) (Σ6 SF) were examined following validated procedures [19]. Body fat percentage (BF%) was calculated using the Yuhasz equation, following the recommendations of the International Society for the Advancement of Kinanthropometry (ISAK) [20]. All participants were measured by the same internationally certified anthropometrist (certificate number: #636739292503670742).

### 2.4. Maximal Oxygen Consumption

For the determination of the maximal O_2_ consumption (VO_2_ max), a modified Bruce treadmill protocol was followed [21]. This test was performed to ensure that all participants were at a similar level of physical condition and that there was homogeneity within the sample.

### 2.5. Dietary Evaluation

A professional registered nutritionist participated in the study (J.M.-A.) and documented the athletes’ daily food and fluid intake throughout the trial. The athletes followed a specific method for dietary recall, which consisted of a food frequency questionnaire (FFQ) that has previously been used for other sports populations [22] being completed at T3. They completed the FFQ to record the “frequency” with which they had consumed 139 different types of food and drink over the previous 8 weeks. The frequency categories were based on the number of times that a food or beverage was consumed per day, week or month and the portion size. The serving sizes were estimated using the standard weight of the food items or using a book that contained over 500 photographs of food [22]. Energy (kcal) and macronutrient (g) consumption was determined by dividing the reported intake by the frequency (in days) using a validated software package (Easy diet^©^, online version 2020, Spanish Academy of Nutrition and Dietetics, Madrid, Spain) [23]. In addition, the total energy intake/kg was calculated for each athlete.

### 2.6. Blood Collection and Analysis

**Figure 1:**
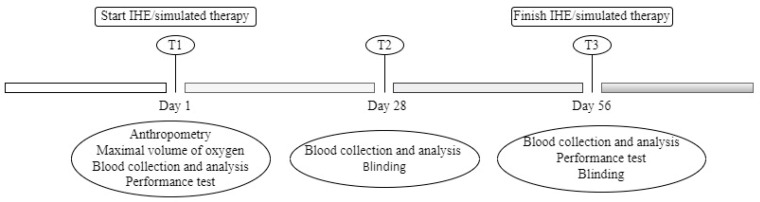
A descriptive diagram of the study timeline.

We followed the World Anti-Doping Agency (WADA) regulations when collecting and transporting the samples [24]. All of our samples were collected under baseline conditions and on an empty stomach, with a period of at least 12 h of fasting since the last meal. All of the blood samples were taken at 08:30 and all of the participants were resting comfortably in a sitting or lying position. The participants were called to the laboratory at 8:30 a.m. on three specific days throughout the study: at the baseline (T1), day 28 (T2), and day 56 (T3) (as shown in Figure 1). The vacutainer system was used (10 mL for serum and 5 mL and 3 mL for EDTA). Immediately after extraction, the tubes were inverted 10 times and were placed in a sealed box to be stored at 4 °C. The temperature during transportation was controlled using a specific label (Libero Ti1, Elpro, Buchs, Switzerland), which was used to measure and register the temperature. The samples were transported under suitable conditions, and they arrived at the laboratory 30 min after extraction. Delays did not affect the analytical quality of the parameters that were studied. The EDTA (anti-coagulant) samples were homogenized for 15 min before being analyzed, as recommended by the WADA.

The tubes that contained blood plus EDTA were centrifuged at 2000 rpm for 15 min. The plasma was extracted using a Pasteur pipette, then transferred to a sterile storage tube and kept at −20 °C until the analysis. The hematological biomarkers of WBC, MON, LYN, RBC, Hb, and Hct were established using a System Coulter Counter MAX-M hematological counter. To determine the EPO biomarker, an immunometric and chemiluminescent trial was conducted in solid phase using the Immulite 2000 EPO analyzer (Diagnostic Products Corporation, Los Angeles CA, USA). The RET biomarker was measured by fluorescence using flow cytometry (Beckman Dickinson, Beckman Coulter). To quantify the contents of the RET-Hb, the XE-2100 analyzer (Sysmex, Mundelein, IL, USA) was used.

Cr and urea were measured using an automatic biochemical analyzer (Cobas^®^ 8000 detection system (775 module); Roche Diagnostics GmbH, Barcelona, Spain). The GFR CDK-EPI was estimated using the equations that were described by Canal et al. [25]. The TP was measured using another automatic analyzer (Hitachi 917, Tokyo, Japan).

The percentage changes in the plasmatic volume (% ΔPV) were calculated using the Van Beaumont equation [26]. Furthermore, the hematological indicator values were adjusted to the changes in the plasmatic volume using the following formula: Corrected value = Uncorrected value × ((100 + % ΔPV)/100) [27].

### 2.7. Performance Tests

The physical performance of the athletes was assessed using individual time trials that were completed at the baseline (T1) and on the final day of the study (T3)., which assessed aerobic power, anaerobic power, and speed over the distances of 60, 400, and 1000 m on an athletics track. The athletics track was 400 m long with eight lanes and was approved by the Royal Spanish Athletics Federation (RFEA). Quadriceps strength was recorded using a dynamometer (Leg Jamar, Chicago, IL, USA).

### 2.8. Blinding

The blinding index was calculated using a questionnaire at T2 and T3. The value of this index ranged from 1 (none of the subjects knew to which group they were assigned) to 0 (all subjects knew to which group they were assigned). Values above 0.5 indicated that the blinding was successful [28].

### 2.9. Statistical Analysis

The study participants were randomly assigned to the HA and NA groups using a stratified block design, which used a sequence that was generated by the “Random Sequence Generator” application according to the date of admission to the study (available at https://apps.apple.com/es/app/random-number-generator-app/id1476396989) (accessed on 5 June 2022). The analyses were performed using STATA version 15.0 (StataCorp, College Station, TX, USA), SPSS software version 24.0 (SPSS, Inc., Chicago, IL, USA), Graphpad Prism (Graphpad Software version 6.01 San Diego, CA, USA), and Microsoft Excel (Microsoft Excel version 19). The data were presented as means and standard deviations and *p* values < 0.05 were considered statistically significant. The Shapiro–Wilk test was used to determine the normality of the variables. Parametric tests were used because the data followed a normal distribution. To determine the differences between the groups (HA and NA) at time points during the study, a *t*-test for independent variables was used for the sample characteristics, dietary assessments, and performance tests. On the other hand, a *t*-test for dependent variables was used to determine the existence of significant differences between the performance tests at T1 and T3. An analysis of variance (ANOVA) with repeated measures was performed to examine the effects of the time × IHE interaction on the different groups (HA and NA) and the hematological behavior, renal function, and sports performance of the groups. The differences between the biomarkers at during times during the study were determined using the Scheffé test. The percentage changes in the performance variables of each group between T1 and T3 were calculated using the following formula: Δ% = ((T3 − T1)/T1) × 100. The *t*-test for independent variables was used to determine whether the differences between these percentages were significant.

### 2.10. Ethical Considerations

The study was approved by the Ethics Committee of the Valladolid East Health Area of University Clinical Hospital of Valladolid (Spain) under CASVE-NM-22-586. All subjects provided written informed consent, in accordance with the Declaration of Helsinki and the 2013 Fortaleza revision (World Medical Association, 2013) [29].

## 3. Results

### 3.1. Recruitment and Randomization

**Figure 2:**
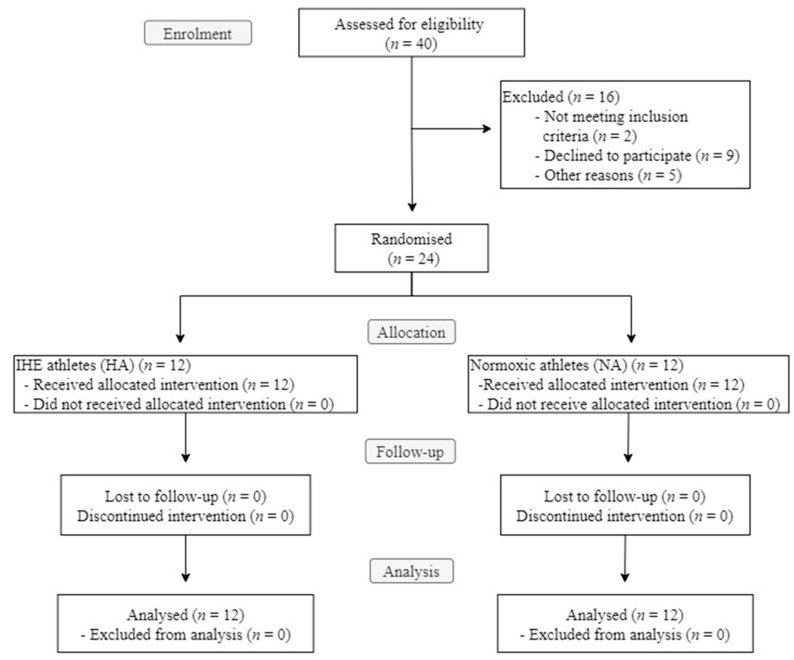
A flow chart of the enrolment and randomization process, according to the CONSORT regulations.

In total, 40 athletes were initially recruited; however, nine athletes decided not to participate in the study. The remaining 31 athletes underwent the VO_2_ max test, of which five were excluded for presenting results that could distort the homogeneity of the sample. These five athletes had just overcome musculoskeletal injuries and were therefore not physically fit enough to follow the training program without risking relapse. In addition, two other athletes were excluded because they were taking drugs that could alter their hematological biomarkers. Overall, 24 athletes were included in the study and were randomly divided into two groups by means of a stratified block design: the hypoxic intervention group (HA; *n* = 12) and the normoxic control group (NA; *n* = 12). None of the 24 participants dropped out of the study or interrupted the intervention, so all participants were tested at T2 and T3 (Figure 2).

### 3.2. Characteristics of the Study Participants

No significant differences (*p* > 0.05) between the evaluated variables among the groups (HA and NA) were observed at the baseline (age, weight, height, Σ6 SF, body fat, and VO_2_ max) (Table 4).

### 3.3. Dietary Evaluation

During the study, there were no statistically significant differences (*p* > 0.05) between the energy, macronutrient, and iron intake of the athletes in the different groups (Table 5).

### 3.4. Biomarkers

The % ΔVP of the participants decreased by 4.5% between T1 and T2 and by 4% between T1 and T3, after adjusting for all of the biomarkers that were analyzed at the different points during the study.

### 3.5. Blood Biomarkers

Table 6 shows the evolution of the biomarkers of hematological behavior at the three time points of the study (T1, T2, and T3) for both groups (HA and NA). No significant differences (*p* > 0.05) between the time points nor between the groups were observed for WBC, MON, LYN, RBC, and Hct. For the HA group, a significant increase (*p* < 0.05) in Hb was observed at T3 (16.15 ± 0.85 g/dL) with respect to T1 (15.21 ± 1.11 g/dL) and with respect to the NA group (14.97 ± 0.92 g/dL). For the HA group, RET and RET-Hb were also significantly increased (*p* < 0.05) at T3 and T2 with respect to T1 and with respect to the NA group. Significant (*p* < 0.05) increases in EPO were observed for the HA group at T2 (6.69 ± 0.88 mU/mL) and T3 (7.05 ± 1.13 mU/mL) with respect to T1 (6.18 ± 1.59 mU/dL) and with respect to the NA group (6.19 ± 1.65 mU/dL). Significant increases in EPO were also seen for the HA group at T3 (7.05 ± 1.13 mU/mL) with respect to T2 (6.69 ± 0.88 mU/mL).

### 3.6. Renal Biomarkers

No significant differences (*p* > 0.05) between the groups nor within the same group at T1, T2 or T3 were observed for urea, GFR CDK-EPI, and TP. The values of Cr in the HA group (1.21 ± 0.03 mg/dL) were significantly (*p* = 0.008) higher than those in the NA group (1.06 ± 0.04 mg/dL), but no significant differences (*p* > 0.05) were found at the different time points of the study within each group (Table 7).

### 3.7. Sports Performance

Table 8 shows the performance test results of the athletes in the HA and NA groups at T1 and T3. Statistically significant improvements (*p* < 0.05) were observed for the HA group in all of the sports performance tests. However, no significant differences (*p* > 0.05) were observed for the NA group. In addition, significant differences between the HA and NA groups were demonstrated in the 400 m test (*p* = 0.003), 1000 m test (*p* < 0.001), 60 m test (*p* < 0.001), and quadriceps strength test (*p* = 0.002).

**Figure 3:**
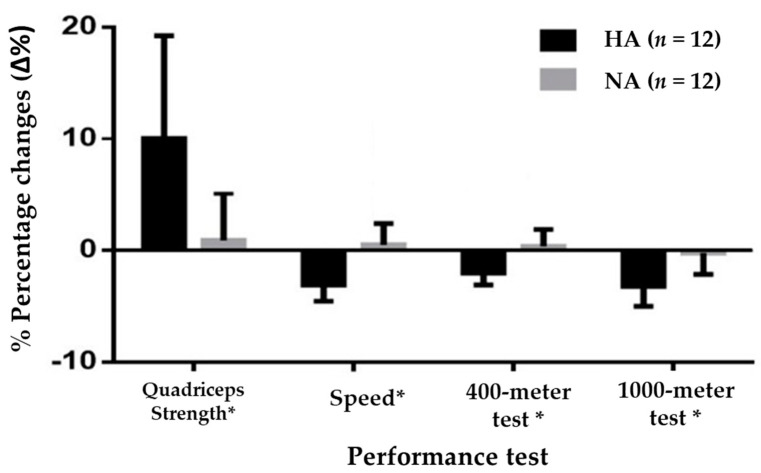
The percentage changes in the performance test results of the two study groups: HA, hypoxic intervention group; NA, control group; % percentage changes (Δ%), ((T3 − T1)/T1) × 100; *, significant differences (*p* < 0.05) between the groups at specific time points, according to the *t*-test for independent variables.

The percentage changes in the performance test results are presented in Figure 3 (quadriceps strength (HA: 10.04 ± 9.21% vs. NA: 0.92 ± 4.22%), speed (HA: −3.09 ± 1.44% vs. NA: 0.51 ± 1.90%), 1000 m test (HA: −3.21 ± 1.80% vs. NA: −0.022 ± 1.91%), and 400 m test (HA: −2.04 ± 1.06% vs. NA: 0.35 ± 1.53%)). Significant differences (*p* < 0.05) were found between the percentages of changes in all tests for the HA group with respect to the NA group.

### 3.8. Blinding Index

The blinding index at T2 was 0.91, with three of the participants stating that they knew the group to which they belonged (although only two of the athletes were correct). The blinding index at T3was 0.87, with three subjects correctly guessing the group to which they belonged. This suggested that the blinding process was successful (Table 9).

## 4. Discussion

The results that were obtained in this study allowed us to partially confirm what we had previously hypothesized. We observed significant increases in some hematological biomarkers (EPO, Hb, RET, and RET-Hb) but no changes in Hct and RBC and a substantial improvement in the performance test results for the HA group with respect to the NA group. Moreover, no disturbances in renal or immunological biomarkers were found that would indicate any danger in the application of the strategy of living and training at 1065 m for 8 weeks, supplemented with IHE.

### 4.1. Blood Biomarkers

Being at altitude causes a reduction in blood O_2_ concentration, which triggers the overexpression of hypoxia inducible factor 1 (*HIF-1*) [30], i.e., an increase in *HIF-1* is inversely related to O_2_ concentration at the cellular level. *HIF-1* regulates the body’s response to hypoxia via a systemic mechanism that is based on the control of O_2_ concentration-sensitive multi-gene expression and the stimulation of the synthesis of the corresponding proteins [31]. One of the genes that increases its expression is the *EPO* gene, which results in the synthesis and release of the erythropoietin protein from the juxtaglomerular cells of efferent arterioles [32,33] after a signaling cascade in the nucleus that involves *CREB-binding protein/p300* transcription factors [34]. Our results showed significant increases in EPO levels when comparing all time points (T2 and T3 vs. T1; T3 vs. T2). These results were in agreement with those from other studies on normobaric IHE [35,36,37,38,39] and hypobaric hypoxia [40,41,42,43] programs that have reported increased EPO concentrations. In contrast, some studies on cyclists [44] and endurance athletes [45] did not observe increased EPO stimulation or improvements in other hematological biomarkers after IHE. These discrepancies between results could be due to the different hypoxic stimuli that were applied (session duration, number of sessions, FIO_2_, simulated altitude, pressure, etc.) or the heterogeneity of the subjects because individual responses to hypoxia develop different patterns of acclimatization to IHE, which must be considered [33].

The process of erythrocyte production (erythropoiesis) takes place in the bone marrow under the control of EPO acting on monopotential cells, which develop into the hematopoietic precursors of RET, with a notable amount of Hb, and become RBC after a maturation period of 2–3 days. The erythrocytosis that is generated by the erythropoietic process, which is induced by IHE, could have the capacity to stimulate the other hemogram variables [11,33]. In fact, the beneficial effects of normobaric IHE protocols that were similar to ours on the RET and Hb levels of rowers [46], cyclists [47], and athletes [48] have been demonstrated and were reproduced in our study. These results were due to the binding of EPO to its receptors, which are located in the medullary erythroid progenitor cells and induce an adequate progression of erythropoietic ontogeny. Furthermore, our athletes presented significant increases in RET-Hb, which indicated that Hb synthesis, iron bioavailability, and medullary activity were optimal during erythropoiesis [49]. Likewise, we observed significant increases in Hb levels between the beginning and the end of the intervention and when comparing participants in the HA group to those in the NA group. Therefore, hypoxic stimuli (IHE) could act through HIF on O_2_-sensitive genes, such as EPO, not only to increase EPO hormone concentration but also to initiate the appropriate development of erythroid cells into RET [32]. Taken together, hypoxic stimuli (living and training at 1065 m plus supplementary IHE) could be potentially effective in accelerating the formation of mature erythrocytes to maintain O_2_ homeostasis.

Different methods for exposure to simulated hypoxia have achieved beneficial hematological adaptations among athletes as increases in Hct, RET, Hb, and HEM have been reported after exposure to IHT [10,48], normobaric IHE [35,36,37,38,39] or hypobaric hypoxia [40,41,42,43]. However, the Hct and HEM biomarkers remained unchanged for the HA group throughout the time points of our study. These results were similar to those from other studies on endurance athletes [45,50], swimmers [51], and cyclists [38,44] that used normobaric IHE. It should be considered that intense and strenuous exercise negatively affects hematological biomarkers, mainly by notably decreasing RBC, Hb, and Hct levels [52,53]. Perhaps the strenuous training loads that were performed in the precompetitive period were responsible for the lack of improvement among some of the biomarkers. Even the intense psychophysical stresses of the athletes have been shown to produce values for hemogram parameters that were below the physiological range, which could be detrimental not only to performance but also to health [38,39]. In this sense, Kasperska et al. [37] and Villa et al. [38] found notable decreases in RET, RBC, Hct, and Hb levels among their control groups (without exposure to hypoxia) with respect to athletes in their IHE intervention groups. Thus, our IHE intervention could exert a protective and moderately stimulating role on erythropoietic and blood biomarkers against the extreme physical demands of elite sport, which are especially increased during precompetitive periods. The modulation or restoration of hematological variables could prevent athletes from overtraining or alterations in their physiological homeostasis, which could negatively influence their sports performance and health. Blood biomarkers have been proposed as suitable indicators for measuring the effects of training and the correct state of health, as well as identifying chronic stress, inflammation, and fatigue [54].

Hypobaric IHE and IHT have greater capacities to stimulate EPO secretion and improve hematological parameters [30]. However, the results that were obtained using our normobaric IHE methodology were similar to those that were obtained from exposure to hypobaric IHE [42] or IHT [10]. This could be because the subjects in the present study lived and trained in Soria (1065 m), which meant that they were exposed to two hypoxic stimuli. In this sense, a study on professional soccer players who lived and trained at 825 m and were supplemented with IHT in comparison to a group of soccer players who completed the same training at sea level [14] reported improvements in EPO, Hb, and Hct levels, which were similar to those that were obtained in the present study. This corroborated the suitability of the employed double hypoxia stimuli methodology, which modulated the strenuous physical loads of exercise and induced benefits on hematological variables.

### 4.2. Sports Performance

Improvements in muscular, blood, cardiovascular, respiratory, and endocrine-metabolic adaptations within the body are directly connected to increases in sports performance, which is the main objective of the application of any programs that involve continuous exposure to hypoxia [39]. Overall, there were improvements of between 3–10% in the results of the performance tests, which were similar to those that were obtained by soccer players who lived and trained at medium altitudes and supplemented their training with a simulated hypoxia situation of 15% FIO_2_ (equivalent to 3000 m) [14]. Although these gains may seem modest, it has been shown that changes of 0.4–0.7% increase the chances of winning an international 1500 m event by 10–20% [45].

Increases in strength after IHT programs have also been reported [55,56]. However, to the best of our knowledge, no evidence of increased strength after IHE has been found until this study [57]. The trigger for improvements in quadriceps strength is probably associated with the increased synthesis and secretion of insulin-like growth factor-1 (IGF-1), testosterone, and the growth hormone that is induced by *HIF-1* [6]. In addition, *HIF-1* activates genes that are involved in angiogenesis, such as vascular endothelial growth factor (VEGF). Therefore, IHE could cause additional vascularization with a greater supply of O_2_ and nutrients and the improved elimination of waste metabolites that, together with muscle hypertrophy and hyperplasia, are responsible for greater contractile efficiency and muscle strength [30,33].

Some normobaric IHE studies [4,10,48,58,59] have shown improvements in time trials (1.5–8%) but not others [45,50,60]. These differences could be due to poor acclimatization to hypoxia that was insufficient to stimulate the optimal physiological responses and the generation of increased stress, which could lead to overtraining or immunological disorders [61]. Our 3% improvements in the 1000 m test were also reported previously [14,62] after exposure to IHE. The adaptations within the hematological biomarkers, the action of *HIF-1* on inducible nitric oxide synthase (*iNOS*) genes, the enhancement of the production of the vasodilator agent NO, and the angiogenic VEGF all result in increased O_2_ delivery to tissues. In addition, increases in the number of mitochondria and the activity of glycolytic pathways, including glucose transporters (GLUTs), generate the predominance of aerobic metabolism and decrease the participation of anaerobic pathways, which allows for the lowering of plasma lactate concentration and delays the onset of fatigue after IHE [30].

The moderate improvements that were observed in the 60 m and 400 m tests could be a consequence of the increased ability to withstand lactate. IHE seems to improve the buffering capacity of muscle [33,63]. In addition, lactate transportation is related to an increased number of MCT1 and MCT4 transporters, which is an adaptation that occurs after adequate acclimatization to hypoxia [64]. An increase in lactate transporters allows for a better exchange and elimination of lactate and, therefore, a slower reduction in pH during exercise, which buffers the acidic state [2,6]. This could have a direct effect on improvements in sports performance. Our results were in agreement with those that were described by Kilding et al. [62] regarding the high-intensity sports performance of basketball players after a 2-week exposure to IHE. Moreover, significant increases in EPO levels could have positive effects on the recovery of athletes due to the protective role of EPO against the free radicals, moderate inflammation, and cell death that are caused by highly demanding physical efforts [65]; therefore, we could speculate that our IHE methodology could influence the recovery process after physical stress and could result in performance improvements.

### 4.3. Safety

Kidneys are sensitive to changes in O_2_ supply, which facilitates the renal production of EPO. However, the accumulation of *HIF-1*, which is induced by IHE, does not protect the kidney against hypoxia and actually accelerates the onset of renal disease by producing fibrosis and glomerular damage [66,67]. Our results for the biomarkers of renal function (urea, Cr, GFR CDK-EPI, and TP) showed no variations after IHE and remained within physiological ranges. Perhaps residing at an average altitude of 1065 m could exert a moderately protective effect on the kidneys because an inverse association has been observed between altitude and all-cause mortality in dialysis patients [68,69], with a 7% decrease in mortality among renal patients who resided between 610 and 1218 m. This could be due to the regulation of enzymes that are induced by hypoxia, which reduces risks for the cardiac and vascular systems. In addition, people who reside at higher altitudes have higher levels of vitamin D [70], which is generally protective against different diseases and mortality [71].

Intense training programs, endurance competitions (such as marathons or long-distance cycling), and exposure to hypoxia (especially IHT) are all forms of extreme physical stress that can cause immunosuppression among athletes, which is associated with increased susceptibility to infections [12,72,73,74]. In our study, no differences were found between the WBC, LYN, and MON levels, which remained at physiological values, at the different time points or among the groups. Our results could imply the adequacy of the physical training program and the IHE methodology. In addition, the athletes living and training at altitude could produce high levels of vitamin D [70], which could modulate their immunological responses [75].

## 5. Conclusions

In summary, the athletes who were living and training at 1065 m and received supplementary IHE sessions were subjected to two hypoxic stimuli, which stimulated significant increases in their blood biomarkers and sports performance. In addition, these two hypoxic stimuli allowed us to improve upon the results that were obtained in previous investigations on normobaric IHE by combining all the benefits that were obtained in other hypobaric IHE or IHT programs with optimal safety profiles.

## 6. Limitations and Strengths

The authors of this review recognized some limitations. Firstly, the small sample size of the study (*n* = 24), which was a consequence of the difficulty in finding professional athletes with similar anthropometric and sporting characteristics who live and train at mid-altitude. Secondly, all participants were male due to the differences that could exist between the biomarker assessments and/or sports performance of athletes of different genders. However, it would be interesting to study the influence of gender on the effects of IHE. Third, we did not perform a follow-up after the end of the 8 weeks of IHE intervention to establish the duration of the adaptations that were obtained.

The main strength of this study was that it was a blinded randomized clinical trial, which allowed us to determine whether there were cause–effect relationships between IHE and changes in blood biomarkers and sports performance tests. In addition, the extraction and collection of the blood samples were performed according to AMA standards and the samples were analyzed in a validated diagnostic laboratory, i.e., the Hematology and Hemotherapy Service of the Hospital de Santa Bárbara, which is a reference public hospital in the province of Soria, Spain.

## 7. Practical Application

This study involved to athletes who participated in the Spanish Athletics Championships in Nerja, Malaga (Spain), in June 2022 and the World Athletics Championships in Eugene, Oregon (United States) in July 2022 and who will participate in the European Athletics Championships in Munich (Germany) in August 2022. In general, IHE plus altitude training could be used prior to high-performance competitions, tournaments, or competitive events to improve hematological biomarkers and sports performance. However, the application of IHE plus altitude training as an ergogenic aid should be considered in light of the sporting objective and the monitoring of the athletes would be necessary. This training program could be applied not only to endurance sports athletes, such as those who compete in athletics, cycling or triathlon, but also to athletes whose competitions are extended over long periods of time and require long and continuous efforts, such as tennis gram slam events or team sports championships (e.g., soccer and basketball, among others).

## Figures and Tables

**Table 1:** The changes that are caused by hypoxia in the different body systems. Adapted with permission from Fernández-Lázaro et al. [2].

Body System	Adaptive Physiological Response to Hypoxia
**Respiratory**	Increase in breathing rate and inspired volume Facilitation of the elimination of CO_2_ Alveolar vasoconstriction Peripheral vasodilatation
**Cardiovascular**	Increase in heart rate and cardiac output Decrease in maximum heart rate Reduction in VO_2_ max Increase in the number and diameter of blood capillaries Decrease in muscular and peripheral vascular resistance Increase in the Borg effect (the difference between the pH of arterial and venous blood) Increase in 2,3-DPG and oxygen release to tissues Decrease in hemoglobin affinity for oxygen
**Endocrine**	Increase in adrenaline, noradrenaline, cortisol, growth hormone, thyroid-stimulating hormone, T3 and T4 hormones, and testosterone Decrease in aldosterone and insulin
**Metabolic**	Utilization of carbohydrates Reference by glycolytic pathways Increase in glycolytic pathway activity Increase in the expression of glucose transporters at the membrane level Facilitation of the control of postprandial blood glucose
**Hematological**	Stimulation of EPO secretion Increase in iron demand Expansion of erythrocyte volume Increase in the volume of red blood cells and blood viscosity
**Immunological**	Acute response: Increase in cardiac output, ventilation, bronchodilation, NK cells, and pro-inflammatory cytokines (such as IL-6) Maintained response: Elevation of IL-6 and increase in monocyte levels
**Muscular**	Increase in oxidative activity, mitochondrial activity, and myoglobin content Changes in aerobic metabolism Increase in volume, strength, and cross-sectional area of muscle fibers

**Abbreviations:** CO_2_, carbon dioxide; VO_2_ max, maximum oxygen volume; 2,3-DPG, 2,3-bisphosphoglyceric acid; T_3_ hormone, triiodothyronine; T_4_ hormone, tetraiodothyronine or tyrosine; EPO, erythropoietin; NK, natural killer cells; IL-6, interleukin-6.

**Table 2:** The main contents of the typical weekly training program that was followed by both groups during the 8 weeks of the study.

Day	Morning	Afternoon
Monday	Lactic Capacity: Interval training series between 200–400 m	Aerobic Capacity: Continuous running for 50–60 min
Tuesday	Aerobic Power: 8–12 km of controlled pace at aerobic threshold	Aerobic Capacity: Continuous running for 50–60 min
Wednesday	Resistance Strength: Interval training on a hill between 200–300 m	Mixed Aerobic–Anaerobic: Fartlek training with changes of pace every 2–5 min for 50–60 min
Thursday	Lactic Power: Interval training series between 300–1000 m	Aerobic Capacity: Continuous running for 50–60 min
Friday	Resistance Speed: Interval training between 100–150 m	Aerobic Capacity: Continuous running for 50–60 min
Saturday	Mixed Aerobic–Anaerobic: Interval training between 1000 and 4000 m	Aerobic Capacity: Continuous running for 50–60 min
Sunday	Aerobic Capacity: Continuous running for between 75–90 min	Rest

**Table 3:** The intermittent hypoxia exposure (IHE) protocol.

Weeks	Duration (Minutes)	FIO_2_ (%)	SaO_2_ (%)	Simulated Altitude (Meters)	Range of Altitude Classification
1–2	90	13	88–84	4000	High altitude
3–4	90	12	84–80	4500	
5–6	90	11	80–78	5000	
7–8	90	10	<78	5500	Very high altitude

**Abbreviations:** FIO_2_, inspired fraction of oxygen; SaO_2_, oxygen saturation.

**Table 4:** The characteristics of the athletes at the baseline of the study.

	HA Athletes	NA Athletes	*p* Value
Sample Size (*n*)	12	12	
Age (years)	26.12 ± 2.90	25.31 ± 4.40	0.619
Body Mass (kg)	63.37 ± 3.72	62.00 ± 4.53	0.353
Height (m)	1.75 ± 0.02	1.73 ± 0.03	0.874
∑ 6 Skinfolds (mm)	32.61 ± 1.50	1.75 ± 0.02	0.561
Body Fat (%)	13.34 ± 0.90	13.75 ± 1.01	0.310
Maximal Oxygen Consumption (mL/kg/min)	57.36 ± 0.96	55.37 ± 1.90	0.417

Data are expressed as mean ± standard deviation. Differences between the groups with *p* values < 0.05 were statistically significant, according to the independent *t*-test.

**Table 5:** The energy, macronutrient, and iron intake of the athletes in the two study groups during the 8 weeks of the study.

	HA Athletes	NA Athletes	*p* Value
Sample Size (*n*)	12	12	
Energy (kcal)	3130 ± 405	3268 ± 358	0.693
Energy (kcal/kg)	49.60 ± 4.30	52.00 ± 5.60	0.126
Protein (g)	151.60 ± 29.40	157.60 ± 2.50	0.786
Protein (%)	17.00 ± 3.20	16.90 ± 2.70	0.318
Protein (g/kg)	2.40 ± 0.70	2.52 ± 0.70	0.830
Animal Protein (g)	83.10 ± 19.30	86.60 ± 24.60	0.531
Vegetal Protein (g)	60.30 ± 19.10	59.30 ± 15.30	0.594
Fat (g)	93.80 ± 20.80	92.60 ± 21.00	0.252
Fat (%)	26.20 ± 4.80	25.60 ± 4.20	0.169
Fat (g/kg)	1.51 ± 0.60	1.48 ± 0.50	0.154
Total Carbohydrates (g)	556.50 ± 58.20	562.20 ± 60.10	0.445
Carbohydrates (%)	64.80 ± 6.10	65.00 ± 5.20	0.320
Carbohydrates (g/kg)	8.82 ± 1.20	9.06 ± 1.30	0.180
Iron (Fe) (mg)	33.00 ± 5.80	32.90 ± 6.10	0.611

Data are expressed as mean ± standard deviation. Differences between the groups with *p* values < 0.05 were statistically significant, according to the independent *t*-test.

**Table 6:** The blood biomarkers of the athletes in the two study groups at the three time points of the study.

Parameter (Reference Value)	Group	Time Point			*p*_1_ Value	*p*_2_ Value
		T1	T2	T3		
**White Blood Cells** **(×10^3^/µL; 3.8–11)**	**HA**	4.77 ± 0.97	4.51 ± 0.97	4.11 ± 1.04	0.774	0.129
	**NA**	5.90 ± 1.50	6.11 ± 1.46	5.86 ± 1.34	0.235	
**Monocytes** **(%; 2.5–10)**	**HA**	7.72 ± 1.01	7.46 ± 1.21	7.47 ± 1.21	0.1364	0.268
	**NA**	6.56 ± 1.67	6.14 ± 1. 13	7.54 ± 2.21	0.409	
**Lymphocytes** **(%; 20–51)**	**HA**	31.11 ± 9.14	31.31 ± 9.06	31.01 ± 8.61	0.124	0.182
	**NA**	31.51 ± 7.50	31.35 ± 7.05	36.98 ± 7.91	0.471	
**Red Blood Cells** **(×10^6^ mL^−1^; 4.5–5.7)**	**HA**	5.32 ± 0.33	5.31 ± 0.34	5.34 ± 0.26	0.354	0.103
	**NA**	5.25 ± 0.40	5.22 ± 0.49	5.27 ± 0.33	0.249	
**Hemoglobin** **(gxdL^−1^; 13–17)**	**HA**	15.21 ± 1.11	15.53 ± 0.97	16.15 ± 0.85 ^a^	<0.05	<0.05
	**NA**	15.33 ± 0.72	15.26 ± 0.85	14.97 ± 0.92	0.89	
**Hematocrit** **(%; 40–50)**	**HA**	45.77 ± 2.79	46.00 ± 2.53	46.14 ± 2.22	0.234	0.127
	**NA**	46.01 ± 2.46	45.69 ± 2.25	45.57 ± 2.51	0.127	
**Reticulocyte Hemoglobin** **(pg; 31.9–41.6)**	**HA**	342.13 ± 10.08	353.12 ± 9.01 ^a^	356.75 ± 10.67 ^a^	< 0.05	<0.05
	**NA**	344.09 ± 7.11	343.09 ± 5.91	342.76 ± 9.13	0.236	
**Reticulocytes** **(%; 0.6–2.4)**	**HA**	1.32 ± 0.33	1.61 ± 0.41 ^a^	1.65 ± 0.47 ^a^	<0.05	< 0.05
	**NA**	1.30 ± 0.27	1.30 ± 0.91	1.29 ± 0.52	0.157	
**Erythropoietin** **(mU/mL; 2.6–18.5)**	**HA**	6.18 ± 1.59	6.69 ± 0.88 ^a^	7.05 ± 1.13 ^a b^	<0.05	<0.05
	**NA**	6.23 ± 1.34	6.21 ± 1.23	6.19 ± 1.65	0.265	

Data are expressed as mean ± standard deviation. Differences between the groups and time points with *p* values < 0.05 were statistically significant, according to the Scheffé test: a, significant difference vs. T1; b, significant difference vs. T2; HA, hypoxic intervention group; NA control group; T1, baseline; T2, day 28; T3, day 56; *p*_1_, the analysis of variance (ANOVA) with repeated measures for each group separately; *p*_2_, the analysis of variance (ANOVA) with repeated measures of two factors to verify the existence of an interaction effect (condition × time).

**Table 7:** The renal biomarkers of the athletes in the two study groups at the three time points of the study.

Parameter (Reference Value)	Group	Time Point			*p*_1_ Value	*p*_2_ Value
		T1	T2	T3		
**Urea (mg/dL; 19–52)**	**HA**	49.55 ± 3.11	50.00 ± 3.07	49.66 ± 3.14	0.994	0.319
	**NA**	37.58 ± 2.01	41.50 ± 1.9	41.75 ± 2.25	0.295	
**Creatinine (mg/dL; 0.8–1.4)**	**HA**	1.20 ± 0.24	1.26 ± 0.22	1.21 ± 0.03	0.164	0.008
	**NA**	1.04 ± 0.37	0.97 ± 0.06	1.06 ± 0.04	0.409	
**Glomerular Filtration (CDK-EPI; > 60)**	**HA**	82.89	78.14	82.06	0.233	0.082
	**NA**	99.18	107.81	96.85	0.471	
**Total Protein (g/dL; 6–8)**	**HA**	7.57 ± 0.31	7.60 ± 0.38	7.67 ± 0.42	0.878	0.660
	**NA**	7.60 ± 0.19	7.64 ± 0.25	7.65 ± 0.98	0.915	

Data are expressed as mean ± standard deviation. Differences between the groups and time points with *p* values < 0.05 were statistically significant, according to the Scheffé test: HA, hypoxic intervention group; NA, control group; T1, baseline; T2, day 28; T3, day 56; *p*_1_, the analysis of variance (ANOVA) with repeated measures for each group separately; *p*_2_, the analysis of variance (ANOVA) with repeated measures of two factors to verify the existence of an interaction effect (condition × time).

**Table 8:** The performance test results of the athletes in the two study groups at T1 and T3.

Performance Test	Group	Time Point		*p* Value
		T1	T3	
**400 m (s)**	**HA ^§^**	55.05 ± 5.78	53.94 ± 5.84	0.003
	**NA**	61.65 ± 5.57 *	60.80 ± 5.13 *	
**1000 m (s)**	**HA ^§^**	155.50 ± 10.88	152.37 ± 9.42	<0.001
	**NA**	159.12 ± 12.67	158.75 ± 12.32	
**Quadriceps Strength (N)**	**HA ^§^**	50.75 ± 19.26	54.38 ± 17.92	0.002
	**NA**	41.13 ± 15.59	41.13 ± 14.75	
**60 m (s)**	**HA ^§^**	8.05 ± 0.97	7.80 ± 0.99	<0.001
	**NA**	8.09 ± 0.98	8.13 ± 0.89	

Data are expressed as mean ± standard deviation: *p*, two-factor repeated measures ANOVA (condition × time); *, significant differences (*p* < 0.05) between the groups at specific time points, according to the *t*-test for independent variables; §, significant differences between T1 and T3 within each group, according to the *t*-test for dependent variables; HA, hypoxic intervention group; NA, control group; T1, baseline; T3, day 56.

**Table 9:** The blinding index for the two study groups at T2 and T3.

Time	No. of Athletes Who Affirmed Their Group	No. of Athletes Who Guessed Their Group Correctly	Blinding Index
**T2**	3	2	0.91
**T3**	3	3	0.87

Data are expressed as mean: T2, day 28; T3, day 56.

## Data Availability

Not applicable.

## Appendix A

**Table A1:** The CONSORT 2010 checklist of information to include when reporting a randomized trial.

Section/Topic	Item Nº	Checklist Item	Reported on Page No.
Title and Abstract	1a	Identification as a randomized trial in the title	1
	1b	A structured summary of the trial design, methods, results, and conclusions (for specific guidance, see CONSORT for abstracts)	2
Introduction			
*Background and Objectives*	2a	The scientific background and an explanation of the rationale	3
	2b	The specific objectives or hypotheses	4
Methods			
*Trial Design*	3a	A description of the trial design (such as parallel, factorial) including allocation ratio	5
	3b	Important changes to the methods after trial commencement (such as eligibility criteria), with reasons	-
*Participants*	4a	The eligibility criteria for the participants	5
	4b	The settings and locations where the data were collected	-
*Interventions*	5	The interventions for each group with sufficient details to allow for replication, including how and when they were actually administered	5–6
*Outcomes*	6a	Completely defined and prespecified primary and secondary outcome measures, including how and when they were assessed	6–8
	6b	Any changes to trial outcomes after the trial commenced, with reasons	-
*Sample Size*	7a	How the sample size was determined	5
	7b	When applicable, an explanation of any interim analyses and stopping guidelines	-
*Randomization*			
Sequence Generation	8a	The method that was used to generate the random allocation sequence	8
	8b	The type of randomization and details of any restrictions (such as blocking and block sizes)	8
Allocation Concealment Mechanism	9	The mechanism that was used to implement the random allocation sequence (such as sequentially numbered containers) and a description of any steps that were taken to conceal the sequence until interventions were assigned	8
Implementation	10	Who generated the random allocation sequence, who enrolled the participants, and who assigned the participants to interventions	8
*Blinding*	11a	When relevant, who was blinded after assignment to interventions (for example, participants, care providers, those assessing outcomes, etc.) and how	6
	11b	When relevant, a description of the similarity of interventions	5–6
*Statistical methods*	12a	The statistical methods that were used to compare groups for primary and secondary outcomes	8–9
	12b	The methods that were used for additional analyses, such as subgroup analyses and adjusted analyses	-
Results			
*Participant Flow* (a diagram is strongly recommended)	13a	The number of participants who were randomly assigned, received intended treatment, and were analyzed for the primary outcome in each group	10 and 35
	13b	The losses and exclusions from each group after randomization, with reasons	10 and 35
*Recruitment*	14a	The dates of the periods of recruitment and follow-ups	7 and 34
	14b	Why the trial ended or was stopped	-
*Baseline Data*	15	A table showing the baseline demographic and clinical characteristics for each group	9 and 28
*Numbers Analyzed*	16	The number of participants (denominator) in each group that were included in each analysis and whether the analysis was performed for the original assigned groups	10 and 35
*Outcomes and Estimation*	17a	The presentation of the results for each primary and secondary outcome for each group and the estimated effect sizes and precision (such as 95% confidence interval)	10–11 and 29–32 and 36
	17b	The presentation of both absolute and relative effect sizes is recommended for binary outcomes	-
*Ancillary Analyses*	18	The results of any other analyses performed, including subgroup analyses and adjusted analyses (distinguishing prespecified from exploratory)	-
*Harm*	19	All significant sources of harm or unintended effects on each group (for specific guidance, see CONSORT for harms)	-
Discussion			
*Limitations*	20	The trial limitations and sources of potential bias, imprecision, and, if relevant, multiplicity of analyses	16
*Generalizability*	21	The generalizability (external validity, applicability, etc.) of the trial findings	12–16
*Interpretation*	22	An interpretation that is consistent with results, a balance of benefits and harm, and a consideration of other relevant evidence	12–16
Other Information			
*Registration*	23	Registration number and name of trial registry	-
*Protocol*	24	Where the full trial protocol can be accessed, when available	-
*Funding*	25	The sources of funding and other support (such as supply of drugs) and the role of funders	-
